# Development and Validation of Nomogram to Preoperatively Predict Intraoperative Cerebrospinal Fluid Leakage in Endoscopic Pituitary Surgery: A Retrospective Cohort Study

**DOI:** 10.3389/fonc.2021.719494

**Published:** 2021-10-26

**Authors:** Xiangming Cai, Junhao Zhu, Jin Yang, Chao Tang, Feng Yuan, Zixiang Cong, Chiyuan Ma

**Affiliations:** ^1^ School of Medicine, Southeast University, Nanjing, China; ^2^ School of Medicine, Nanjing University, Nanjing, China; ^3^ Department of Neurosurgery, Jinling Hospital, Nanjing, China; ^4^ School of Nanjing Medicine, Southern Medical University, Guangzhou, China

**Keywords:** albumin, cerebrospinal fluid leakage, endoscopic pituitary surgery, nomogram, pituitary tumors, tumor height

## Abstract

**Background:**

Pituitary adenomas (PAs) are the most common tumor of the sellar region. PA resection is the preferred treatment for patients with clear indications for surgery. Intraoperative cerebrospinal fluid (iCSF) leakage is a major complication of PA resection surgery. Risk factors for iCSF leakage have been studied previously, but a predictive nomogram has not yet been developed. We constructed a nomogram for preoperative prediction of iCSF leakage in endoscopic pituitary surgery.

**Methods:**

A total of 232 patients who underwent endoscopic PA resection at the Department of Neurosurgery in Jinling Hospital between January of 2018 and October of 2020 were enrolled in this retrospective study. Patients treated by a board-certified neurosurgeon were randomly classified into a training cohort or a validation cohort 1. Patients treated by other qualified neurosurgeons were included in validation cohort 2. A range of demographic, clinical, radiological, and laboratory data were acquired from the medical records. The Least Absolute Shrinkage and Selection Operator (LASSO) algorithm and uni- and multivariate logistic regression were utilized to analyze these features and develop a nomogram model. We used a receiver operating characteristic (ROC) curve and calibration curve to evaluate the predictive performance of the nomogram model.

**Results:**

Variables were comparable between the training cohort and validation cohort 1. Tumor height and albumin were included in the final prediction model. The area under the curve (AUC) of the nomogram model was 0.733, 0.643, and 0.644 in training, validation 1, and validation 2 cohorts, respectively. The calibration curve showed satisfactory homogeneity between the predicted probability and actual observations. Nomogram performance was stable in the subgroup analysis.

**Conclusions:**

Tumor height and albumin were the independent risk factors for iCSF leakage. The prediction model developed in this study is the first nomogram developed as a practical and effective tool to facilitate the preoperative prediction of iCSF leakage in endoscopic pituitary surgery, thus optimizing treatment decisions.

## Introduction

Pituitary adenomas (PAs) comprise approximately 15% of primary intracranial neoplasms, and comprehensive management of PAs includes transsphenoidal surgical resection, radiotherapy, and medications (1). Endoscopic transsphenoidal surgery is a highly effective first-line treatment for PAs. However, there are many potential complications in this surgical approach (2). Intraoperative cerebrospinal fluid (iCSF) leakage is one of the major complications and remains a major driver of postoperative CSF leakage and meningitis (3, 4). Moreover, iCSF leakage leads to more thorough and meticulous reconstruction strategies and impacts postoperative management (5).

The preoperative prediction for iCSF leakage is valuable and could allow for improved patient counseling and impact surgical plans. Victor et al. proposed a machine learning (ML) model based on clinical and radiological data which performed well, achieving an AUC of 0.84 and an accuracy of 88% (4). However, applying this ML model in actual practice requires supporting software and platforms, which have not been assessed so far (6).

Nomogram is an easy-to-use predictive tool with user-friendly graphical interfaces, providing visualization of complex statistical predictive models (7). Nomograms have been widely utilized to predict both binary and prognostic outcomes (7). However, there is no nomogram for preoperatively predicting iCSF leakage during endoscopic pituitary surgery in patients with PAs. Therefore, we aimed to construct and validate the first predictive nomogram for preoperatively forecasting iCSF leakage during endoscopic pituitary surgery in patients with PAs.

## Methods

### Patients Selection and Data Collection

We reviewed the clinical records of PA patients who underwent endoscopic PA resection at the Neurosurgery Department in Jinling Hospital between January of 2018 and October of 2020. The inclusion criteria were as follows: (1) pathologically confirmed PA, (2) patients who underwent PA resection *via* transsphenoidal endoscopic approach, (3) patients with a clear surgery record regarding iCSF leakage, and (4) patients who had at least one collected variable. The exclusion criteria were: (1) patients without histopathological examination, and (2) patients who had no collected variables. This retrospective study was approved by our institutional research ethics board (2021NZKY-037-01). Informed consent was waived because of the data anonymization before analysis and the retrospective nature of the investigation.

Overall, 96 items were collected in this study. Clinical characteristics collected from the medical records included age, gender, primary-recurrence subtype, treatment history for PAs (medication, surgery, and radiotherapy), and preoperative signs and symptoms (moon face, acromegalia, headache, visual impairment, and visual field defect). Included patients were also diagnosed with clinical subtypes including nonfunctioning, growth hormone (GH) secreting (8, 9), prolactin (PRL) secreting (9), and adrenocorticotropic hormone (ACTH) secreting (10) PAs. We also collected information on radiological features including tumor size (tumor volume and lengths of tumor maximum dimension, height, width, and thickness), the minimum intercarotid distance at the horizontal C4 segment of the internal carotid artery (ICDC4h) (11), Knosp grade, Hardy grade, tumor shape 1 (in sella, ellipsoid, or hourglass signs), tumor shape 2 (lobulated shape), tumor signal intensity (T2-weighted magnetic resonance imaging (MRI) signal intensity compared with white matter), sellar barrier (12) (strong or weak), multiple lesions, optic nerve compression, and pituitary apoplexy. Grades 0*–*2 and grades 3*–*4 were classified into noninvasive and invasive classes, respectively, for Knosp grade (13) and Hardy grade for sellar invasion (14). We extracted 74 variables from preoperative laboratory tests, including pituitary hormones, routine blood work, coagulation, renal and hepatic functions, and electrolytes, which were based on preoperative peripheral blood samples (**Supplementary Table S2**). The outcome, iCSF leakage, was extracted from the surgical records.

### Nomogram Development and Validation

First, the patients treated by a board-certified neurosurgeon (CY-M) were randomly divided into a training cohort and a validation cohort 1. Patients treated by other qualified neurosurgeons were included in a validation cohort 2. Then, included variables were submitted to a least absolute shrinkage and selection operator (LASSO) algorithm to filter features missing < 60% of data using the “glmnet” R package (version 4.1). Mean imputation was utilized for missing data only during the LASSO analysis. Missing data were not imputed in the following analyses to simulate model performance in real-world conditions. Finally, independent risk factors associated with iCSF leakage were identified with uni- and multivariate logistic regressions and visualized as a nomogram using the “rms” R package (version 6.1.0). To evaluate the model’s predictive performance, the receiver operating characteristic (ROC) curve and the calibration curve were computed separately with “pROC” (version 1.17.0.1) and “rms” R packages. We also conducted subgroup analysis on validation cohorts to assess the robustness of the model performance according to age, gender, primary-recurrence subtype, clinical subtype, Knosp and Hardy grades, and characteristics included in the final model. The mean values of continuous variables in the validation cohort 1 were used as the cutoff values in the subgroup analysis.

### Statistical Analysis

The processes of model construction and validation in the current study were carried out according to “Transparent Reporting of a Multivariable Prediction Model for Individual Prognosis or Diagnosis” (TRIPOD) guidance (**Supplementary Table S1**) (15). Due to a lack of generally accepted sample size estimation techniques for risk prediction models, we applied the events per variable (EVP) = 10 criteria (16). Based on the criteria, the event number in the training dataset needs to exceed 10 × the number of variables included in the multivariate regression analysis. Although there were 21 variables processed into the regression analysis, we only analyzed all possible combinations up to five variables in the multivariate logistic regression analysis. As there are 51 events in the training cohort, the sample size was sufficient for this research.

Continuous data were expressed as the mean ± standard deviation (SD). We used Student’s *t*-test to compare two continuous variables and the chi-squared test or Fisher’s exact test for comparisons between categorized variables. Spearman correlation analysis was used to evaluate the relationship between two continuous variables, and the results were visualized with “ggplot” (version 3.3.3) R packages. The R software (version 3.6.0) was applied for these statistical analyses, and *p* < 0.05 was considered statistically significant.

## Results

### Baseline Patient Characteristics

A total of 158 eligible patients, treated by a board-certified neurosurgeon (CY-M), were randomly divided into the training cohort (n = 119) and the validation cohort 1 (n = 39). Another 74 patients, treated by other qualified neurosurgeons, were enrolled into validation cohort 2 according to the inclusion and exclusion criteria. Detailed baseline characteristics for the training cohort and the validation cohort 1 were summarized in **Supplementary Table S2** and showed homogeneity in these cohorts. The baseline characteristics of samples with and without iCSF leakage (**Table 1** and **Supplementary Table S3**) revealed significant differences in lengths of tumor height and thickness, tumor volume, triiodothyronine (T3), tumor shape 1, tumor shape 2, and sellar barrier.

**Table 1:** Important characteristics of patients in the without iCSF leakage group and in the with iCSF leakage group.

Characteristics	Without iCSF leakage	With iCSF leakage	*p*
Gender			0.060
Female	41 (47.1%)	45 (63.4%)	
Male	46 (52.9%)	26 (36.6%)	
Primary-recurrence subtype			0.227
Primary	78 (89.7%)	58 (81.7%)	
Recurrence	9 (10.3%)	13 (18.3%)	
Lengths of tumor maximum dimension (mm)	24.69 ± 8.44	28.62 ± 11.4	0.092
Lengths of tumor height (mm)	20.48 ± 8.31	26.3 ± 12.05	0.006*
Lengths of tumor width (mm)	21.76 ± 5.37	24.07 ± 8.64	0.208
Lengths of tumor thickness (mm)	18.1 ± 5.93	22.14 ± 8.76	0.009*
Tumor volume (mm^3^)	4.88 ± 4.23	10.18 ± 11.31	0.016*
Tumor shape 1			0.025*
In sella	13 (19.4%)	5 (9.3%)	
Hourglass sign	27 (40.3%)	35 (64.8%)	
Ellipsoid	27 (40.3%)	14 (25.9%)	
Tumor shape 2			0.019*
Not lobulated	63 (94%)	42 (77.8%)	
Lobulated	4 (6%)	12 (22.2%)	
Sellar barrier			0.042*
Weak	18 (26.9%)	25 (46.3%)	
Strong	49 (73.1%)	29 (53.7%)	
Knosp grade			0.192
Noninvasive	56 (65.9%)	38 (54.3%)	
Invasive	29 (34.1%)	32 (45.7%)	
Pituitary apoplexy			0.335
No	66 (76.7%)	48 (68.6%)	
Yes	20 (23.3%)	22 (31.4%)	
Acromegalia			0.701
No	73 (83.9%)	57 (80.3%)	
Yes	14 (16.1%)	14 (19.7%)	
TSH (mIU/L)	1.83 ± 1.18	2.37 ± 1.94	0.151
T3 (nmol/L)	1.21 ± 0.35	1.31 ± 0.31	0.049*
T4 (nmol/L)	95.22 ± 22.88	100.72 ± 25	0.173
Monocyte percentage (%)	7.43 ± 1.75	6.96 ± 1.32	0.098
APTT (s)	26.67 ± 3.77	27.43 ± 3.37	0.206
Fibrinogen (g/L)	2.64 ± 0.68	2.73 ± 0.75	0.537
Albumin (g/L)	40.33 ± 3.69	39.65 ± 4.12	0.351

TSH, thyroid-stimulating hormone; T3, triiodothyronine; T4, tetraiodothyronine; APTT, activated partial thromboplastin time; iCSF, intraoperative cerebrospinal fluid. *Statistical significance.

### Filtering Process for Collected Variables

**Figure 1:**
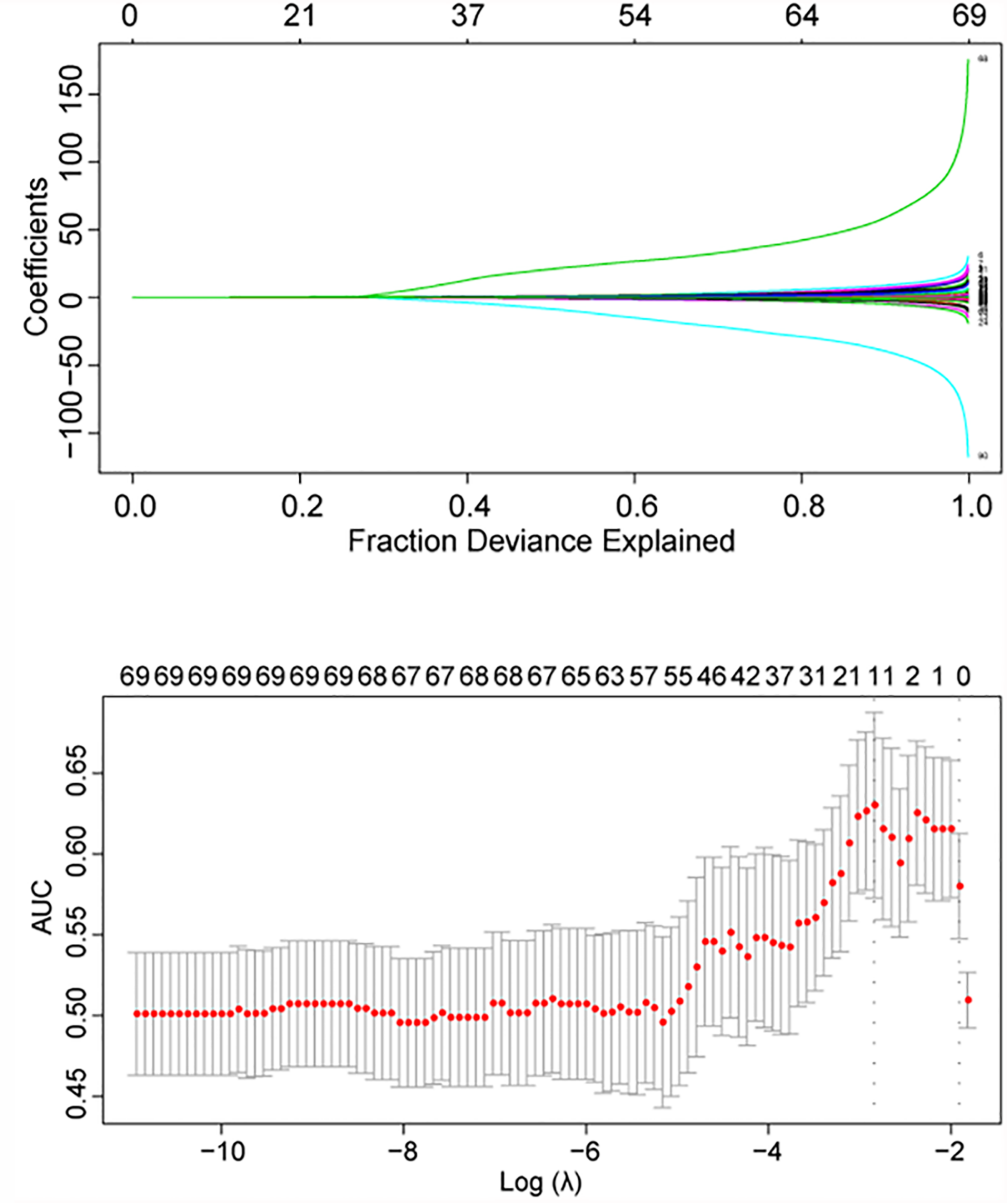
LASSO regression analysis using 10-fold cross-validation. AUC, area under the curve.

In a univariate logistic regression analysis, the Hardy grade for suprasellar extension, tumor shape 1, Knosp grade, tumor shape 2, sellar barrier, lengths of tumor maximum dimension, height, width and thickness, and tumor volume were predictive factors with *p* < 0.05 (**Supplementary Table S4**). Among these variables, only the sellar barrier was a protective factor for iCSF leakage, whereas the other factors were all risk factors. We then conducted the LASSO analysis, and the following 15 features were screened out of the original 87 (**Supplementary Table S5** and **Figure 1**): Hardy grade for suprasellar extension, gender, acromegalia, pituitary apoplexy, tumor shape 2, lengths of tumor height and thickness, tumor volume, thyroid-stimulating hormone (TSH), T3, tetraiodothyronine (T4), albumin, monocyte percentage, activated partial thromboplastin time (APTT), and fibrinogen.

### Independent Predictors for iCSF leakage

After the filtering process, a total of 21 variables were screened out by univariate analysis and LASSO analysis. These variables were further analyzed in a multivariable logistic regression analysis. We analyzed all possible combinations of up to five variables in the multivariate logistic regression analysis. Finally, lengths of tumor height (odds ratio (OR), 95% Confidence Interval (CI): 1.1141, 1.0485*–*1.1839, *p* = 0.0005) and albumin (OR, 95% CI: 0.8698, 0.7576*–*0.9986, *p* = 0.0477) were incorporated into the multivariate model, as shown in **Table 2**.

**Table 2:** Univariate and multivariate logistic regression analysis for the final model.

Characteristics	Univariate analysis				Multivariate analysis			
	Coefficient	OR	95% CI	*p*	Coefficient	OR	95% CI	*p*
Lengths of tumor height (mm)	0.0768	1.0798	1.0299-1.1321	0.0015*	0.1081	1.1141	1.0485-1.1839	0.0005*
Albumin (g/L)	-0.0757	0.9271	0.8304-1.0350	0.1779	-0.1395	0.8698	0.7576-0.9986	0.0477*

CI, confidence interval; OR, odds ratio. *Statistical significance.

### Development and Validation of the Nomogram

**Figure 2:**
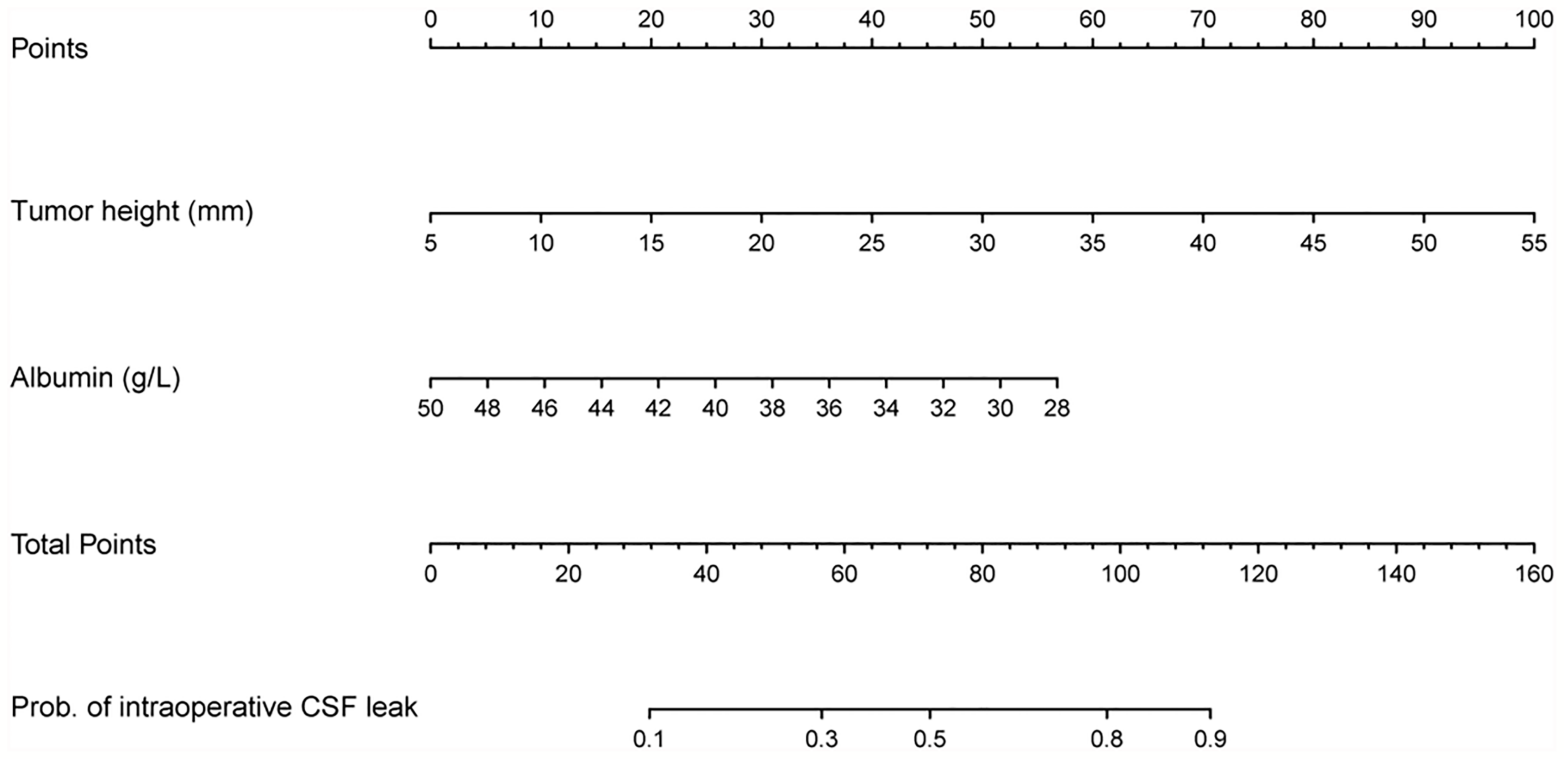
Nomogram for preoperatively predicting the proportion of iCSF leakage during endoscopic pituitary surgery in patients with pituitary tumor.

**Figure 3:**
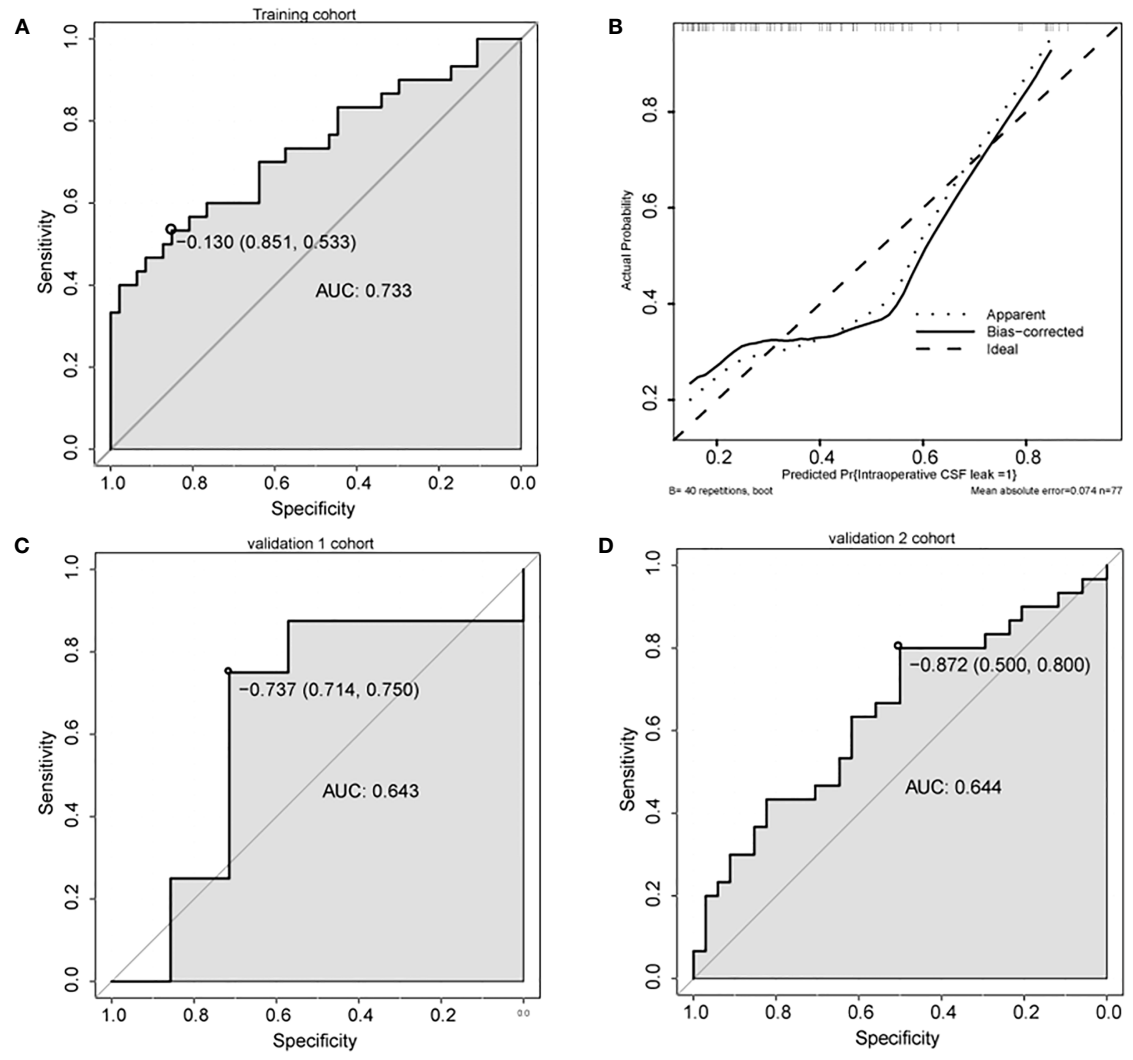
Predictive performance of the nomogram. **(A, C, D)** ROC analysis of nomogram in training cohort **(A)**, validation cohort 1 **(C)**, and validation cohort 2 **(D)**. **(B)** Calibration plots of the nomogram. AUC, area under the curve.

A nomogram was constructed based on the multivariate model (**Figure 2**). For each patient, users need to draw virtual vertical lines from each variable to the “Points” axis, identify the points for each variable, and sum these scores to calculate the total point. Then, users should compare the total point with the probability scale to evaluate the probability of iCSF leakage. The areas under the curve (AUCs) of the training cohort, validation cohort 1, and validation cohort 2 were 0.733, 0.643, and 0.644, respectively (**Figures 3A, C, D**). A calibration curve was generated showing adequate prediction accuracy using this model (**Figure 3B**). Subgroup analysis revealed the nomogram had stable predictive performance in validation cohort 1 (**Supplementary Figure S1** and **Table 3**) and validation cohort 2 (**Supplementary Figure S2** and **Table 3**).

**Table 3:** ROC analysis in subgroups from validation cohort 1 and 2.

Subgroups	AUC 1	AUC 2
Age		
>51 years	0.562	0.627
≤51 years	0.625	0.647
Gender		
Female	0.417	0.587
Male	0.844	0.705
Clinical subtype		
Nonfunctioning	0.611	0.625
PRL secreting	NA	0.600
GH secreting	0.750	0.714
ACTH secreting	NA	NA
Primary-recurrence subtype		
Primary	0.708	0.619
Recurrence	0.500	0.829
Maximum dimension		
Microadenoma (<10 mm)	NA	0.500
Macroadenoma (10–40 mm)	0.727	0.669
Giant adenoma (≥40 mm)	NA	0.867
Knosp grade		
Noninvasive	0.600	0.571
Invasive	0.800	0.789
Hardy grade for sellar invasion		
Noninvasive	0.675	0.631
Invasive	0.611	0.500
Lengths of tumor height		
>23.6mm	0.667	0.611
≤23.6mm	0.500	0.490
Albumin		
>41g/L	0.562	0.627
≤41g/L	0.750	0.647

PRL secreting, prolactin secreting; GH secreting, growth hormone secreting; ACTH secreting, adrenocorticotropic hormone secreting. AUC 1, area under curve for validation cohort 1; AUC 2, area under curve for validation cohort 2; NA, Not applicable.

## Discussion

PAs account for approximately 15% of primary intracranial and central nervous system tumors (1). Endoscopic transsphenoidal surgery is the preferred treatment for patients with clear indications for surgery. iCSF leakage is one of the major complications of PA resection surgery (3, 4). Preoperative prediction of iCSF leakage could assist neurosurgeons in developing individualized surgery strategies for patients with PAs. However, no user-friendly predictive tool for preoperatively predicting iCSF leakage is available. In the current research, lengths of tumor height and albumin were identified as independent predictive factors for iCSF leakage. These two variables were incorporated into a nomogram model to preoperatively calculate the probability of iCSF leakage tailored to individual patients.

A series of risk factors for iCSF leakage have been identified. Karnezis et al. found that in endoscopic sellar surgery, craniopharyngioma, mild liver disease, and extension into the anterior cranial fossa are preoperative risk factors for iCSF leakage (17). Patel et al. revealed that body mass index, suprasellar extension, hydrocephalus, and craniopharyngioma are significant independent predictors for iCSF leakage in endoscopic transsphenoidal sellar surgery (18). Zhou et al. discovered that tumor consistency (*p* = 0.001; OR = 2.379) and tumor size (*p* = 0.026; OR = 1.032) were independent predictors for iCSF leakage in endoscopic transsphenoidal PA surgery (19). Wang et al. found that, in endoscopic transsphenoidal PA surgery, tumor diameter was the independent predictor for iCSF leakage (*p* = 0.004, OR = 1. 090) (20). Liu et al. proposed that applying intraoperative lumbar drainage (LD) significantly decreased the iCSF leakage rate (10.1% *vs*. 31.4%, *p* < 0.001) in endoscopic transsphenoidal surgery of pituitary macroadenomas (21).

The protective role of LD was supported by a meta-analysis conducted by Tan et al. (22). Xue et al. discovered that tumors with lobular or irregular contours and gonadotrophic-positive staining increased the risk of iCSF leakage in endoscopic transsphenoidal PA surgery (23). We also found that tumors with a lobulated shape are a risk factor for iCSF leakage in univariate logistic regression analysis (*p* = 0.0383; OR = 3.712; **Supplementary Table S4**). However, this variable was filtered out in the multivariate analysis. Villalonga et al. classified the sellar barrier into strong, mixed, and weak, based on the relationship between the pituitary gland, tumor, and CSF (12). They found that a strong sellar barrier significantly reduced the iCSF leakage rate (RR = 0.08; 95% CI 0.03-0.19; *p* < 0.0001), while a weak sellar barrier was associated with higher rates of iCSF leakage (RR = 8.54; 95% CI 5.4-13.5; *p* < 0.0001).

We also investigated the relationship between the sellar barrier and iCSF leakage. However, we only separated the sellar barrier into two types: strong or weak. Strong denoted normal pituitary tissue exists between PAs and CSF, which included strong and mixed sellar barriers, as described by Villalonga et al. Weak is the weak sellar barrier described by Villalonga et al. The protective effect of a strong sellar barrier for iCSF leakage was detected in the current research in a univariate logistic regression analysis (*p* = 0.0427; OR = 0.413; **Supplementary Table S4**). However, this effect disappeared in the multivariate logistic regression analysis.

Victor et al. proposed the only ML model for predicting iCSF leakage during endoscopic transsphenoidal surgery for PAs (4). Although they analyzed a series of clinical and radiological variables, no factor showed significant predictive value for iCSF leakage in traditional uni- or multivariate analyses. However, with the same variables, the ML model they constructed had an AUC of 0.84 in the validation dataset. In our work, we witnessed a range of variables with significant predictive effect in the comparison between groups with and without iCSF leakage [lengths of tumor height and thickness, tumor volume, T3, tumor shape 1, tumor shape 2, and sellar barrier (**Table 1**)] and univariate logistic regression analysis [Hardy grade for suprasellar extension, tumor shape 1, Knosp grade, tumor shape 2, sellar barrier, lengths of tumor maximum dimension, height, width and thickness, and tumor volume (**Supplementary Table S4**)] We combined these variables with other variables screened out from the LASSO analysis to conduct a multivariable logistic regression analysis.

The final model included two independent predictors for iCSF leakage, which were lengths of tumor height (OR, 95% CI: 1.1141, 1.0485*–*1.1839, *p* = 0.0005) and albumin (OR, 95% CI: 0.8698, 0.7576*–*0.9986, *p* = 0.0477). Compared with the ML model constructed by Victor et al., our model had relatively lower AUCs (0.643 and 0.644 for validation cohorts 1 and 2, respectively) but better interpretability. Clinicians can distinguish the risk predictor from the protective predictor based on the OR value. The application of the ML model remains a challenge in clinical practice because there is no easy-to-use platform for prediction based on the ML model (6). After the multivariate regression model was visualized into a nomogram, our model was clinically useful and easy to incorporate into clinical practice.

As mentioned above, some research has found a significant relationship between iCSF leakage and preoperative factors, including suprasellar extension (18), tumor size (19), tumor diameter (20), lobular tumors (23), and sellar barrier (12). These factors also showed significant predictive value in our univariate analysis (**Supplementary Table S4**). However, all of them were filtered out in the multivariate regression analysis, and only lengths of tumor height remained in the final model, which seems to be related to these filtered variables. We conducted correlation analyses between length of tumor height and some of these filtered variables (**Supplementary Table S6** and **Supplementary Figure S3**). The results showed that length of tumor height was significantly correlated with tumor volume (R = 0.87; *p* < 0.01; **Supplementary Figure S3B**), length of tumor maximum dimension (R = 0.93; *p* < 0.01; **Supplementary Figure S3A**), length of tumor width (R = 0.83; *p* < 0.01; **Supplementary Figure S3D**), and length of tumor thickness (R = 0.88; *p* < 0.01; **Supplementary Figure S3C**). We also discovered that length of tumor height differed significantly between groups according to the Knosp grade (*p* < 0.001; **Supplementary Table S6**), Hardy grade for suprasellar extension (*p* < 0.001; **Supplementary Table S6**), tumor shape 1 (*p* < 0.001; **Supplementary Table S6**), tumor shape 2 (*p* = 0.017; **Supplementary Table S6**), and sellar barrier (*p* = 0.002; **Supplementary Table S6**). Based on these results, we suggest that there may be collinearity between length of tumor height and these factors.

We found that albumin was an independent protective predictor for iCSF leakage (OR, 95% CI: 0.8698, 0.7576*–*0.9986, *p* = 0.0477; **Table 2**). This result was supported by Karnezis et al. Their research examined 1,108 people with pituitary adenomas and 53 people with craniopharyngiomas who underwent endoscopic sellar surgery, and mild liver disease was revealed to be a risk factor for iCSF leakage (OR=3.636, *p* =0.046), which usually lead to low albumin levels. Based on the review by Wang et al., lower albumin levels function as a biomarker for immune dysfunction (24), and Zhang et al. discovered that invasive PAs showed higher infiltration of M2-like tumor-associated macrophages (25), which have an anti-inflammatory phenotype (26). We suggest that lower albumin levels may act as a biomarker for an anti-inflammatory immune environment in PAs, which is suggestive of invasive tumor behavior. However, this hypothesis needs to be verified in further research.

There were several limitations in this study. First, because of the lack of thyrotropinoma and gonadotropinoma patients in the center, we only included nonfunctioning, GH secreting, PRL secreting, and ACTH secreting PAs. This may have caused potential selection bias, which is unavoidable in a single-institution retrospective study. Therefore, further studies are needed to comprehensively evaluate the nomogram model. Second, the model’s predictive performance was unsatisfactory, however, this is the first nomogram for preoperative prediction of iCSF leakage in endoscopic pituitary surgery for PA patients. Its predictive performance in some subgroups was relatively sufficient, and we recommend that clinicians apply this model in carefully selected patients. For example, patients that are male (AUCs = 0.844, 0.705 for validation cohort 1 and 2, respectively), with GH secreting PAs (AUCs = 0.750, 0.714 for validation cohort 1 and 2, respectively), and with an invasive Knosp grade (AUCs = 0.800, 0.789 for validation cohort 1 and 2, respectively) are appropriate for this nomogram. Third, some variables extracted in this study were missing data. However, all of the variables processed into the LASSO and univariate regression analyses had less than 60% of their data missing. Furthermore, only 35.3% of data in the final model was missing, which is acceptable and provides an adequate sample size for the multivariate analysis. Finally, because the current work focused on all patients with PAs treated with endoscopic pituitary surgery, nomograms for particular subgroup populations were not computed in this research. Additional research is warranted to calculate the predictive nomogram model for iCSF leakage for various subgroup populations.

## Conclusions

This study revealed that tumor height and albumin were independent risk factors associated with iCSF leakage. Albumin is found for the first time to be an independent predictor for iCSF leakage. This study developed and validated a feasible and stable novel nomogram for preoperative prediction of iCSF leakage, which could assist neurosurgeons in developing individualized operation plans for patients with PAs. This may optimize treatment results.

## Data Availability Statement

The raw data of this article is available from the corresponding author upon reasonable request.

## Ethics Statement

The studies involving human participants were reviewed and approved by Research ethics board of Jinling Hospital. The ethics committee waived the requirement of written informed consent for participation.

## Author Contributions

CM conceived and designed the investigation. XC analyzed the data and drafted the manuscript. JZ, JY, CT, FY, and ZC conducted statistical analyses. All authors have read and approved the manuscript.

## Conflict of Interest

The authors declare that the research was conducted in the absence of any commercial or financial relationships that could be construed as a potential conflict of interest.

## Publisher’s Note

All claims expressed in this article are solely those of the authors and do not necessarily represent those of their affiliated organizations, or those of the publisher, the editors and the reviewers. Any product that may be evaluated in this article, or claim that may be made by its manufacturer, is not guaranteed or endorsed by the publisher.

## Supplementary Material

Supplementary Figure 1 Predictive performance for the nomogram in the subgroup analysis from validation cohort 1. **(A, B)** ROC analysis in the subgroup according to age (**A** for >51 years and **B** for ≤51 years); **(C, D)** ROC analysis in the subgroup according to gender (**C** for female and **D** for male); **(E, F)** ROC analysis in the subgroup according to clinical subtypes (**E** for nonfunctioning and **F** for GH secreting); **(G, H)** ROC analysis in the subgroup according to primary-recurrence subtypes (**G** for primary and **H** for recurrence subtypes); **(I)** ROC analysis in the subgroup according to the maximum dimension (**I** for macroadenoma); **(J, K)** ROC analysis in the subgroup according to Knosp grade (**J** for noninvasive and **K** for invasive); **(L, M)** ROC analysis in the subgroup according to Hardy grade (**L** for noninvasive and **M** for invasive); **(N, O)** ROC analysis in the subgroup according to length of tumor height (**N** for >23.6 mm and **O** for ≤23.6 mm); **(P, Q)** ROC analysis in the subgroup according to albumin (**P** for >41g/L and **Q** for ≤41g/L).

Supplementary Figure 2 Predictive performance for the nomogram in the subgroup analysis from validation cohort 2. **(A, B)** ROC analysis in the subgroup according to age (**A** for >51 years and **B** for ≤51 years); **(C, D)** ROC analysis in the subgroup according to gender (**C** for female and **D** for male); **(E–G)** ROC analysis in the subgroup according to clinical subtypes (**E** for nonfunctioning, **F** for PRL secreting and **G** for GH secreting); **(H, I)** ROC analysis in the subgroup according to primary-recurrence subtypes (**H** for primary and **I** for recurrence subtypes); **(J–L)** ROC analysis in the subgroup according to the maximum dimension (**J** for microadenoma, **K** for macroadenoma and **L** for giant adenoma); **(M, N)** ROC analysis in the subgroup according to Knosp grade (**M** for noninvasive and **N** for invasive); **(O, P)** ROC analysis in the subgroup according to Hardy grade (**O** for noninvasive and **P** for invasive); **(Q, R)** ROC analysis in the subgroup according to length of tumor height (**Q** for >23.6 mm and **R** for ≤23.6 mm); **(S, T)** ROC analysis in the subgroup according to albumin (**S** for >41g/L and **T** for ≤41g/L).

Supplementary Figure 3 Correlation analysis. Correlation between length of tumor height and length of tumor maximum dimension **(A)**, tumor volume **(B)**, length of tumor thickness **(C)**, and length of tumor width **(D)**, respectively.
