# Supplementary figures and images for: Development and Validation of Nomogram to Preoperatively Predict Intraoperative Cerebrospinal Fluid Leakage in Endoscopic Pituitary Surgery: A Retrospective Cohort Study
Source: Front Oncol. 2021 Oct 26;11:719494. doi: 10.3389/fonc.2021.719494 (PMC8576331; doi:10.3389/fonc.2021.719494)

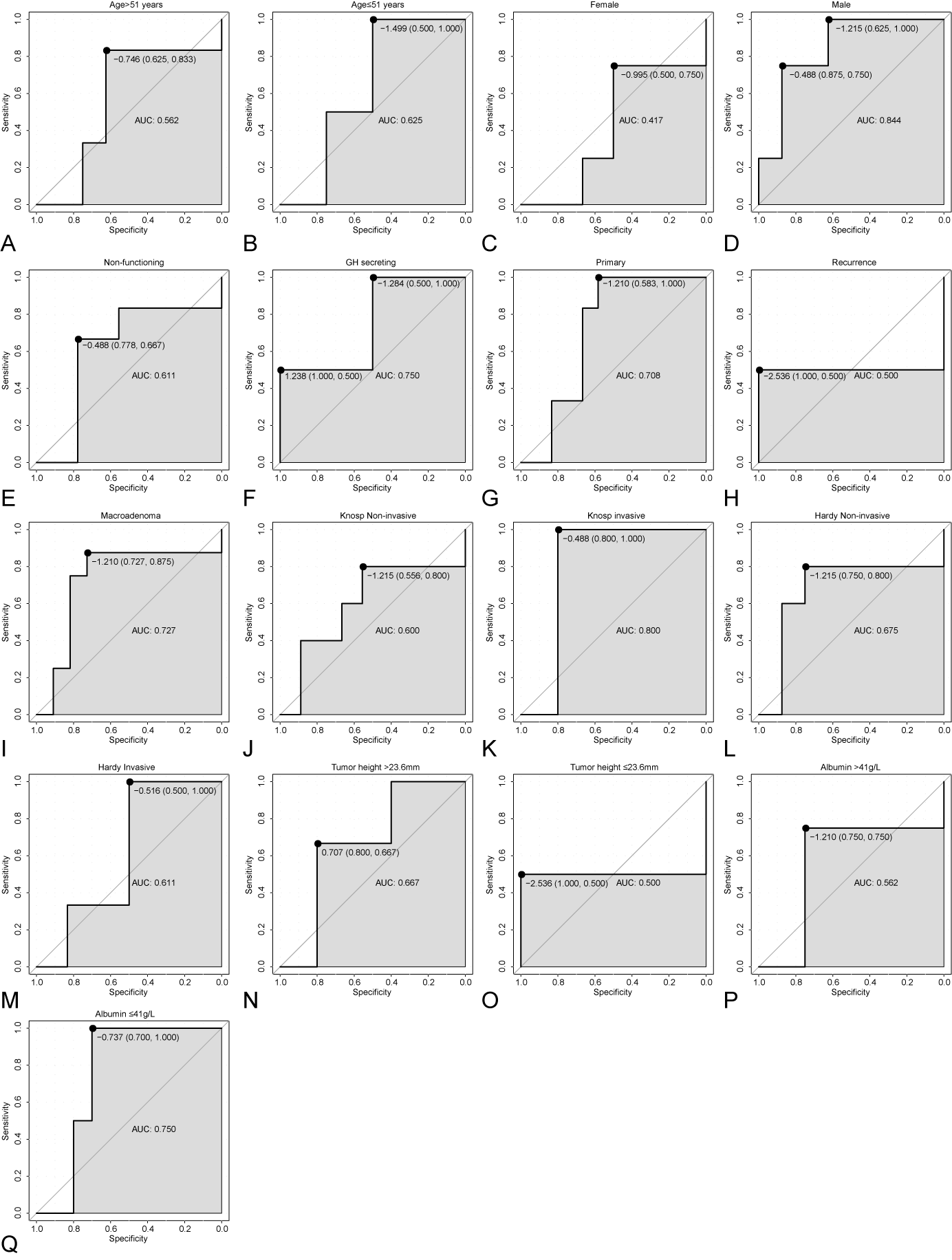

Supplement: Supplementary Figure 1 — Predictive performance for the nomogram in the subgroup analysis from validation cohort 1. (A, B) ROC analysis in the subgroup according to age (A for >51 years and B for ≤51 years); (C, D) ROC analysis in the subgroup according to gender (C for female and D for male); (E, F) ROC analysis in the subgroup according to clinical subtypes (E for nonfunctioning and F for GH secreting); (G, H) ROC analysis in the subgroup according to primary-recurrence subtypes (G for primary and H for recurrence subtypes); (I) ROC analysis in the subgroup according to the maximum dimension (I for macroadenoma); (J, K) ROC analysis in the subgroup according to Knosp grade (J for noninvasive and K for invasive); (L, M) ROC analysis in the subgroup according to Hardy grade (L for noninvasive and M for invasive); (N, O) ROC analysis in the subgroup according to length of tumor height (N for >23.6 mm and O for ≤23.6 mm); (P, Q) ROC analysis in the subgroup according to albumin (P for >41g/L and Q for ≤41g/L). [file Image_1.tif]

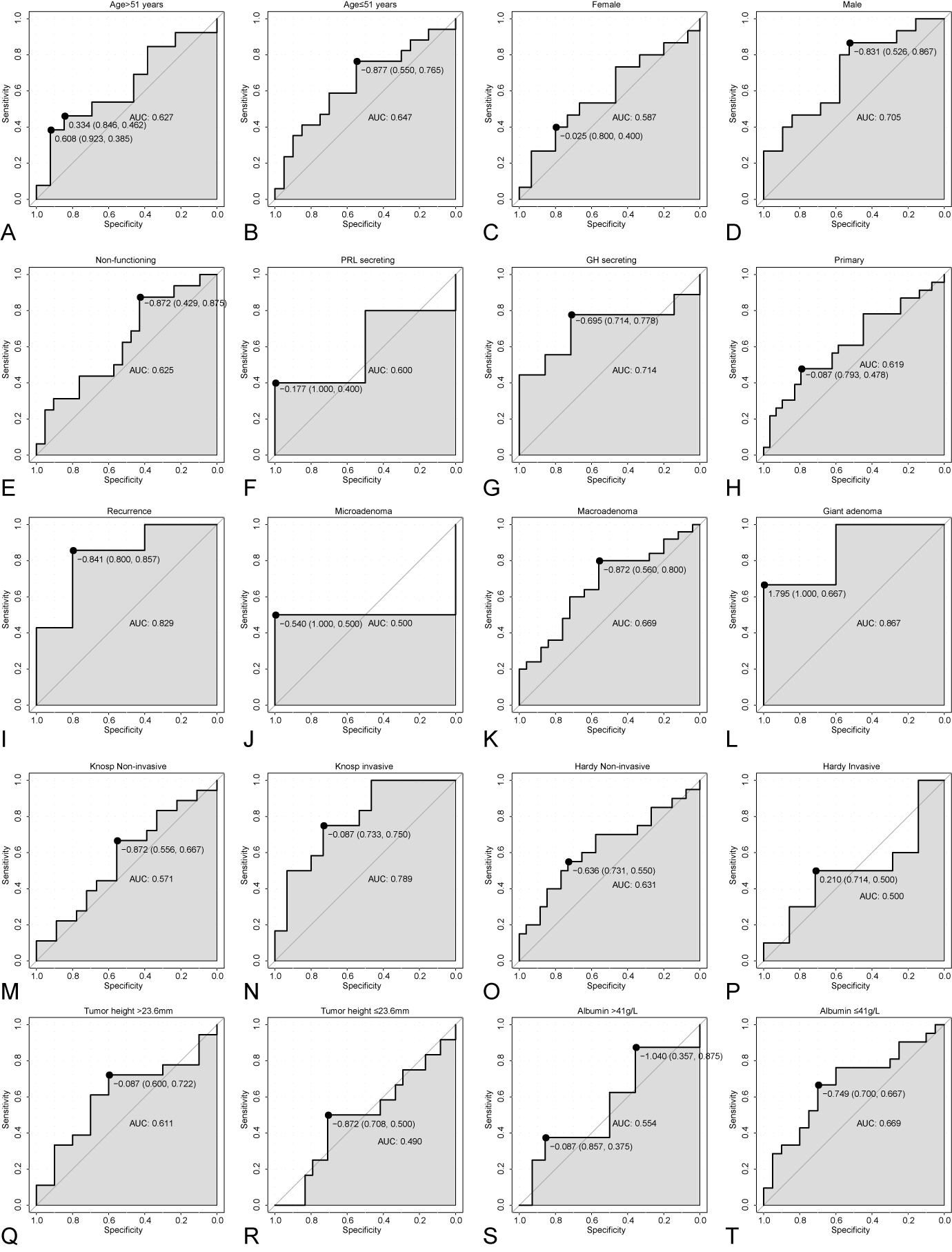

Supplement: Supplementary Figure 2 — Predictive performance for the nomogram in the subgroup analysis from validation cohort 2. (A, B) ROC analysis in the subgroup according to age (A for >51 years and B for ≤51 years); (C, D) ROC analysis in the subgroup according to gender (C for female and D for male); (E–G) ROC analysis in the subgroup according to clinical subtypes (E for nonfunctioning, F for PRL secreting and G for GH secreting); (H, I) ROC analysis in the subgroup according to primary-recurrence subtypes (H for primary and I for recurrence subtypes); (J–L) ROC analysis in the subgroup according to the maximum dimension (J for microadenoma, K for macroadenoma and L for giant adenoma); (M, N) ROC analysis in the subgroup according to Knosp grade (M for noninvasive and N for invasive); (O, P) ROC analysis in the subgroup according to Hardy grade (O for noninvasive and P for invasive); (Q, R) ROC analysis in the subgroup according to length of tumor height (Q for >23.6 mm and R for ≤23.6 mm); (S, T) ROC analysis in the subgroup according to albumin (S for >41g/L and T for ≤41g/L). [file Image_2.tif]

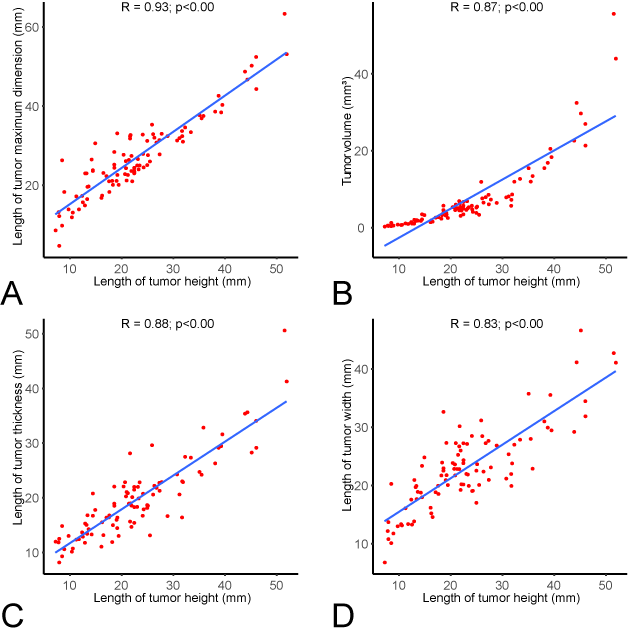

Supplement: Supplementary Figure 3 — Correlation analysis. Correlation between length of tumor height and length of tumor maximum dimension (A), tumor volume (B), length of tumor thickness (C), and length of tumor width (D), respectively. [file Image_3.tif]
