# Supplementary material for: Development and Validation of Nomogram to Preoperatively Predict Intraoperative Cerebrospinal Fluid Leakage in Endoscopic Pituitary Surgery: A Retrospective Cohort Study
Source: Front Oncol. 2021 Oct 26;11:719494. doi: 10.3389/fonc.2021.719494 (PMC8576331; doi:10.3389/fonc.2021.719494)
Supplement: Supplementary file 5 [file Table_2.docx]

Supplementary Table 2. Characteristics of patients with PA in the training cohort and validation cohort 1

| Characteristics | Training cohort | Validation cohort 1 | *p* |
| --- | --- | --- | --- |
| Intraoperative CSF leakage |  |  | 0.464 |
| No | 68 (57.1%) | 19 (48.7%) |  |
| Yes | 51 (42.9%) | 20 (51.3%) |  |
| Age (year) | 52.25±12.81 | 50.97±12.74 | 0.368 |
| Gender |  |  | 0.400 |
| Female | 62 (52.1%) | 24 (61.5%) |  |
| Male | 57 (47.9%) | 15 (38.5%) |  |
| Primary-recurrence subtype |  |  | 0.620 |
| Primary | 101 (84.9%) | 35 (89.7%) |  |
| Recurrence | 18 (15.1%) | 4 (10.3%) |  |
| Clinical subtype |  |  | 0.134 |
| Nonfunctioning | 85 (71.4%) | 24 (60%) |  |
| PRL secreting | 5 (4.2%) | 6 (15%) |  |
| GH secreting | 26 (21.8%) | 9 (22.5%) |  |
| ACTH secreting | 3 (2.5%) | 1 (2.5%) |  |
| Lengths of tumor maximum dimension (mm) | 26.56±10.31 | 26.33±9.34 | 0.799 |
| Lengths of tumor height (mm) | 22.92±10.31 | 23.66±11.45 | 0.903 |
| Lengths of tumor width (mm) | 22.94±7.16 | 22.23±6.87 | 0.898 |
| Lengths of tumor thickness (mm) | 19.71±7.26 | 20.73±8.82 | 0.663 |
| Tumor volume (mm^3^) | 7.21±9.00 | 7.48±6.97 | 0.841 |
| ICDC4h | 1.62±0.63 | 1.54±0.39 | 0.972 |
| Hardy grade for suprasellar extension |  |  | 0.887 |
| 0 | 27 (23.1%) | 7 (18.4%) |  |
| A | 27 (23.1%) | 11 (28.9%) |  |
| B | 36 (30.8%) | 11 (28.9%) |  |
| C | 20 (17.1%) | 8 (21.1%) |  |
| D | 4 (3.4%) | 0 (0%) |  |
| E | 3 (2.6%) | 1 (2.6%) |  |
| Hardy grade for sellar invasion |  |  | 0.414 |
| Noninvasive | 87 (74.4%) | 25 (65.8%) |  |
| Invasive | 30 (25.6%) | 13 (34.2%) |  |
| Knosp grade |  |  | 0.348 |
| Noninvasive | 68 (58.1%) | 26 (68.4%) |  |
| Invasive | 49 (41.9%) | 12 (31.6%) |  |
| Tumor shape 1 |  |  | 0.909 |
| In sella | 14 (14.6%) | 4 (16%) |  |
| Hourglass sign | 50 (52.1%) | 12 (48%) |  |
| Ellipsoid | 32 (33.3%) | 9 (36%) |  |
| Tumor shape 2 |  |  | 0.520 |
| Not lobulated | 82 (85.4%) | 23 (92%) |  |
| Lobulated | 14 (14.6%) | 2 (8%) |  |
| Sellar barrier |  |  | 1.000 |
| Weak | 34 (35.4%) | 9 (36%) |  |
| Strong | 62 (64.6%) | 16 (64%) |  |
| Tumor signal intensity |  |  | 0.768 |
| Lower | 13 (13.8%) | 4 (16%) |  |
| Equal | 54 (57.4%) | 16 (64%) |  |
| Higher | 27 (28.7%) | 5 (20%) |  |
| Multiple lesions |  |  | 1.000 |
| No | 115 (98.3%) | 38 (100%) |  |
| Yes | 2 (1.7%) | 0 (0%) |  |
| Optic nerve compression |  |  | 0.846 |
| No | 41 (35%) | 12 (31.6%) |  |
| Yes | 76 (65%) | 26 (68.4%) |  |
| Pituitary apoplexy |  |  | 0.759 |
| No | 85 (72%) | 29 (76.3%) |  |
| Yes | 33 (28%) | 9 (23.7%) |  |
| History of pituitary surgery |  |  | 0.936 |
| No | 98 (82.4%) | 33 (84.6%) |  |
| Yes | 21 (17.6%) | 6 (15.4%) |  |
| History of medication |  |  | 0.151 |
| No | 118 (99.2%) | 37 (94.9%) |  |
| Yes | 1 (0.8%) | 2 (5.1%) |  |
| History of radiotherapy |  |  | 1.000 |
| No | 118 (99.2%) | 39 (100%) |  |
| Yes | 1 (0.8%) | 0 (0%) |  |
| Headache |  |  | 0.132 |
| No | 64 (53.8%) | 27 (69.2%) |  |
| Yes | 55 (46.2%) | 12 (30.8%) |  |
| Visual impairment |  |  | 0.549 |
| No | 52 (44.1%) | 20 (51.3%) |  |
| Yes | 66 (55.9%) | 19 (48.7%) |  |
| Visual field defect |  |  | 0.260 |
| No | 78 (65.5%) | 30 (76.9%) |  |
| Yes | 41 (34.5%) | 9 (23.1%) |  |
| Moon face |  |  | 1.000 |
| No | 117 (98.3%) | 38 (97.4%) |  |
| Yes | 2 (1.7%) | 1 (2.6%) |  |
| Acromegalia |  |  | 1.000 |
| No | 98 (82.4%) | 32 (82.1%) |  |
| Yes | 21 (17.6%) | 7 (17.9%) |  |
| Prolacin (mIU/L) | 516.22±679.08 | 694.93±945.41 | 0.509 |
| Testosterone (nmol/L) | 4.97±5.51 | 3.98±4.39 | 0.843 |
| Estradiol (pmol/L) | 126.38±102.05 | 238.82±317.06 | 0.127 |
| Progesterone (nmol/L) | 2.45±5.26 | 6.01±13.48 | 0.315 |
| LH (IU/L) | 6.31±8.50 | 4.59±5.00 | 0.160 |
| FSH (IU/L) | 16.84±20.04 | 14.09±18.25 | 0.304 |
| DHEAS (umol/L) | 3.57±2.45 | 3.59±2.71 | 0.837 |
| TSH (mIU/L) | 2.08±1.68 | 2.07±1.30 | 0.599 |
| T3 (nmol/L) | 1.26±0.35 | 1.24±0.30 | 0.821 |
| T4 (nmol/L) | 98.00±25.18 | 96.88±20.00 | 0.785 |
| FT3 (pmol/L) | 4.36±0.80 | 4.34±0.73 | 0.929 |
| FT4 (pmol/L) | 9.78±2.78 | 9.62±1.95 | 0.938 |
| ACTH (pg/ml) | 31.43±25.87 | 25.34±14.02 | 0.144 |
| Cortisol (μmol/L) | 0.31±0.16 | 0.30±0.14 | 0.678 |
| IGF-1 (ng/ml) | 234.16±287.08 | 422.75±325.26 | 0.123 |
| IGFBP3 (mg/L) | 5.18±2.30 | 6.09±2.34 | 0.147 |
| GH (μg/L) | 3.32±8.01 | 3.00±8.56 | 0.797 |
| RBC count (10^12^/L) | 4.44±0.48 | 4.40±0.47 | 0.673 |
| HCT (%) | 0.40±0.04 | 0.40±0.04 | 0.898 |
| RDW (%) | 13.05±1.21 | 12.90±0.57 | 0.765 |
| MCV (fL) | 89.59±5.51 | 90.51±4.32 | 0.213 |
| MCH (pg) | 30.10±2.09 | 30.42±2.07 | 0.200 |
| Hemoglobin (g/L) | 133.31±15.02 | 133.65±16.17 | 0.910 |
| MCHC (g/L) | 336.04±12.83 | 335.78±13.51 | 0.679 |
| WBC count (10^9^/L) | 5.65±1.58 | 5.90±1.64 | 0.297 |
| Neutrophil percentage (%) | 53.75±10.35 | 55.00±8.46 | 0.422 |
| Lymphocyte percentage (%) | 36.23±9.55 | 35.15±8.02 | 0.500 |
| Monocyte percentage (%) | 7.30±1.63 | 6.99±1.45 | 0.279 |
| Basophil percentage (%) | 0.40±0.24 | 0.40±0.28 | 0.527 |
| Eosinophil percentage (%) | 2.33±1.31 | 2.46±2.10 | 0.418 |
| Platelet count (10^9^/L) | 202.50±55.00 | 210.73±47.73 | 0.384 |
| Thrombocytocrit (%) | 0.21±0.05 | 0.23±0.05 | 0.152 |
| MPV (fL) | 10.77±1.16 | 10.98±1.41 | 0.245 |
| APTT (s) | 27.02±3.56 | 27.01±3.76 | 0.980 |
| TT (s) | 17.04±1.14 | 17.32±1.20 | 0.223 |
| PT (s) | 11.36±0.78 | 11.17±0.63 | 0.133 |
| Antithrombin III (%) | 88.86±15.02 | 87.57±16.22 | 0.499 |
| FDP (μg/mL) | 2.17±2.88 | 1.90±0.74 | 0.607 |
| Fibrinogen (g/L) | 2.69±0.72 | 2.63±0.68 | 0.532 |
| Total protein (g/L) | 68.80±6.11 | 69.98±5.27 | 0.380 |
| Albumin (g/L) | 39.76±3.86 | 41.00±3.77 | 0.114 |
| Globulin (g/L) | 29.05±3.72 | 28.98±2.64 | 0.899 |
| ALT (U/L) | 33.04±18.63 | 32.89±19.09 | 0.775 |
| AST (U/L) | 25.34±11.4 | 26.44±12.91 | 0.865 |
| ALP (U/L) | 74.67±23.83 | 72.59±20.31 | 0.977 |
| LDH (U/L) | 430.07±137.96 | 478.14±111.87 | 0.306 |
| Total cholesterol (mmol/L) | 4.49±0.97 | 4.94±0.71 | 0.098 |
| TG (mmol/L) | 2.05±1.31 | 2.07±1.30 | 0.797 |
| Total bilirubin (μmol/L) | 13.36±6.14 | 13.22±5.35 | 0.807 |
| Unconjugated bilirubin (μmol/L) | 9.03±5.95 | 8.27±4.99 | 0.702 |
| Calcium (mmol/L) | 2.39±0.11 | 2.41±0.13 | 0.301 |
| Potassium (mmol/L) | 4.07±0.35 | 4.14±0.32 | 0.099 |
| Chlorine (mmol/L) | 102.51±2.99 | 103.59±3.18 | 0.028 |
| Sodium (mmol/L) | 141.00±3.09 | 141.27±3.00 | 0.972 |
| CK-MB isoenzyme (U/L) | 6.62±6.70 | 8.43±10.80 | 0.865 |
| PCT (μg/L) | 0.04±0.02 | 0.05±0.02 | 0.066 |
| CRP (mg/L) | 1.96±5.64 | 3.87±12.85 | 0.294 |
| D-dimer (mg/L) | 0.35±0.87 | 0.32±0.18 | 0.062 |
| IL-6 (ng/L) | 4.5±3.17 | 8.26±22.73 | 0.412 |
| INR | 0.99±0.07 | 0.97±0.06 | 0.122 |
| Creatinine (μmol/L) | 60.91±15.98 | 56.53±10.56 | 0.126 |
| Urea (mmol/L) | 5.22±1.34 | 4.96±1.35 | 0.321 |
| Uric acid (μmol/L) | 309.95±86.82 | 304.51±94.54 | 0.766 |
| Glucose (mmol/L) | 5.24±1.83 | 5.65±3.53 | 0.428 |
| Total carbon dioxide (mmol/L) | 25.96±3.14 | 27.23±2.18 | 0.169 |

PRL secreting, prolactin secreting; GH secreting, growth hormone secreting; ACTH secreting, adrenocorticotropic hormone secreting; ICDC4h, the minimum intercarotid distance at the horizontal C4 segment of the internal carotid artery; LH, luteinizing hormone; FSH, follicle-stimulating hormone; DHEAS, dehydroepiandrosterone sulfate; TSH, thyroid-stimulating hormone; T3, triiodothyronine; T4, tetraiodothyronine; FT3, free triiodothyronine; FT4, free tetraiodothyronine; ACTH, adrenocorticotropic hormone; IGF-1, insulin-like growth factor-1; IGFBP3, insulin-like growth factor binding protein 3; GH, growth hormone; RBC, red blood cell; HCT, haematocrit; RDW, red blood cell distribution width; MCV, mean corpuscular volume; MCH, mean corpuscular hemoglobin; MCHC, mean corpuscular hemoglobin concentration; WBC, white blood cell; MPV, mean platelet volume; APTT, activated partial thromboplastin time; TT, thrombin time; PT, prothrombin time; FDP, fibrin/fibrinogen degradation products; ALT, alanine aminotransferase; AST, aspartate transaminase; ALP, alkaline phosphatase; LDH, lactate dehydrogenase; TG, triglyceride; PCT, procalcitonin; CRP, C-reactive protein; IL-6, interleukin-6; INR, international normalized ratio. Tumor signal intensity: T2-weighted magnetic resonance imaging signal intensity of tumor compared with that of white matter.
