# Supplementary material for: Development and Validation of Nomogram to Preoperatively Predict Intraoperative Cerebrospinal Fluid Leakage in Endoscopic Pituitary Surgery: A Retrospective Cohort Study
Source: Front Oncol. 2021 Oct 26;11:719494. doi: 10.3389/fonc.2021.719494 (PMC8576331; doi:10.3389/fonc.2021.719494)
Supplement: Supplementary file 6 [file Table_3.docx]

Supplementary Table 3. Other characteristics of patients in the without iCSF leakage group and in the with iCSF leakage group

| Characteristics | Without iCSF leakage | With iCSF leakage | *p* |
| --- | --- | --- | --- |
| Age (year) | 51.63±12.84 | 52.31±12.76 | 0.716 |
| Clinical subtype |  |  | 0.960 |
| Nonfunctioning | 60 (67.4%) | 49 (70%) |  |
| PRL secreting | 7 (7.9%) | 4 (5.7%) |  |
| GH secreting | 20 (22.5%) | 15 (21.4%) |  |
| ACTH secreting | 2 (2.2%) | 2 (2.9%) |  |
| ICDC4h | 1.59±0.62 | 1.62±0.55 | 0.457 |
| Hardy grade for suprasellar extension |  |  | 0.210 |
| 0 | 23 (27.1%) | 11 (15.7%) |  |
| A | 23 (27.1%) | 15 (21.4%) |  |
| B | 25 (29.4%) | 22 (31.4%) |  |
| C | 11 (12.9%) | 17 (24.3%) |  |
| D | 1 (1.2%) | 3 (4.3%) |  |
| E | 2 (2.4%) | 2 (2.9%) |  |
| Hardy grade for sellar invasion |  |  | 0.977 |
| Noninvasive | 62 (72.9%) | 50 (71.4%) |  |
| Invasive | 23 (27.1%) | 20 (28.6%) |  |
| Tumor signal intensity |  |  | 0.789 |
| Lower | 9 (13.8%) | 8 (14.8%) |  |
| Equal | 40 (61.5%) | 30 (55.6%) |  |
| Higher | 16 (24.6%) | 16 (29.6%) |  |
| Multiple lesions |  |  | 1.000 |
| No | 84 (98.8%) | 69 (98.6%) |  |
| Yes | 1 (1.2%) | 1 (1.4%) |  |
| Optic nerve compression |  |  | 0.131 |
| No | 34 (40%) | 19 (27.1%) |  |
| Yes | 51 (60%) | 51 (72.9%) |  |
| Headache |  |  | 1.000 |
| No | 50 (57.5%) | 41 (57.7%) |  |
| Yes | 37 (42.5%) | 30 (42.3%) |  |
| Visual impairment |  |  | 0.103 |
| No | 45 (52.3%) | 27 (38%) |  |
| Yes | 41 (47.7%) | 44 (62%) |  |
| Visual field defect |  |  | 0.084 |
| No | 65 (74.7%) | 43 (60.6%) |  |
| Yes | 22 (25.3%) | 28 (39.4%) |  |
| Moon face |  |  | 0.588 |
| No | 86 (98.9%) | 69 (97.2%) |  |
| Yes | 1 (1.1%) | 2 (2.8%) |  |
| History of pituitary surgery |  |  | 0.315 |
| No | 75 (86.2%) | 56 (78.9%) |  |
| Yes | 12 (13.8%) | 15 (21.1%) |  |
| History of medication |  |  | 0.253 |
| No | 84 (96.6%) | 71 (100%) |  |
| Yes | 3 (3.4%) | 0 (0%) |  |
| History of radiotherapy |  |  | 0.449 |
| No | 87 (100%) | 70 (98.6%) |  |
| Yes | 0 (0%) | 1 (1.4%) |  |
| Prolacin (mIU/L) | 580.57±735.54 | 535.71±780.54 | 0.282 |
| Testosterone (nmol/L) | 4.97±5.32 | 4.43±5.22 | 0.714 |
| Estradiol (pmol/L) | 140.97±127.43 | 170.64±239.78 | 0.855 |
| Progesterone (nmol/L) | 3±6.58 | 3.82±10 | 0.376 |
| LH (IU/L) | 6.57±8.97 | 5.07±6.08 | 0.430 |
| FSH (IU/L) | 16.93±22.01 | 15.22±16.24 | 0.892 |
| DHEAS (umol/L) | 3.65±2.62 | 3.48±2.34 | 0.850 |
| FT3 (pmol/L) | 4.37±0.75 | 4.33±0.81 | 0.716 |
| FT4 (pmol/L) | 9.85±2.26 | 9.61±2.95 | 0.302 |
| ACTH (pg/ml) | 27.43±16.89 | 32.93±29.57 | 0.124 |
| Cortisol (μmol/L) | 0.31±0.17 | 0.3±0.14 | 0.827 |
| IGF-1 (ng/ml) | 258.68±314.67 | 275.99±287.65 | 0.559 |
| IGFBP3 (mg/L) | 5.63±2.55 | 4.97±1.94 | 0.372 |
| GH (μg/L) | 3.15±8.1 | 3.36±8.2 | 0.421 |
| RBC count (10^12^/L) | 4.44±0.44 | 4.41±0.52 | 0.764 |
| HCT (%) | 0.4±0.04 | 0.4±0.04 | 0.819 |
| RDW (%) | 12.98±1.14 | 13.05±1.03 | 0.522 |
| MCV (fL) | 89.71±5.05 | 89.94±5.52 | 0.644 |
| MCH (pg) | 30.17±2.2 | 30.19±1.93 | 0.546 |
| Hemoglobin (g/L) | 133.7±14.69 | 133±16.05 | 0.782 |
| MCHC (g/L) | 336.17±14.17 | 335.75±11.34 | 0.610 |
| WBC count (10^9^/L) | 5.67±1.71 | 5.76±1.45 | 0.616 |
| Neutrophil percentage (%) | 53.01±10.04 | 55.36±9.66 | 0.147 |
| Lymphocyte percentage (%) | 36.76±9.25 | 34.96±9.06 | 0.232 |
| Basophil percentage (%) | 0.42±0.27 | 0.37±0.21 | 0.356 |
| Eosinophil percentage (%) | 2.37±1.47 | 2.35±1.62 | 0.859 |
| Platelet count (10^9^/L) | 208.54±50.9 | 199.48±56.1 | 0.306 |
| Thrombocytocrit (%) | 0.22±0.05 | 0.21±0.05 | 0.204 |
| MPV (fL) | 10.79±1.17 | 10.87±1.3 | 0.672 |
| TT (s) | 17.16±1.26 | 17.05±1.02 | 0.479 |
| PT (s) | 11.3±0.68 | 11.33±0.82 | 0.737 |
| Antithrombin III (%) | 89.32±15.83 | 87.71±14.66 | 0.752 |
| FDP (μg/mL) | 2.45±3.4 | 1.72±0.8 | 0.359 |
| Total protein (g/L) | 69.35±5.98 | 68.68±5.85 | 0.420 |
| Globulin (g/L) | 29.03±3.43 | 29.03±3.57 | 0.899 |
| ALT (U/L) | 32.87±16.96 | 33.18±20.8 | 0.574 |
| AST (U/L) | 24.71±10.42 | 26.83±13.33 | 0.660 |
| ALP (U/L) | 76.39±27.6 | 71.33±15.12 | 0.668 |
| LDH (U/L) | 445.33±61.98 | 445.38±149.08 | 0.999 |
| Total cholesterol (mmol/L) | 4.52±0.75 | 4.73±1.07 | 0.460 |
| TG (mmol/L) | 1.87±1.14 | 2.24±1.42 | 0.526 |
| Total bilirubin (μmol/L) | 13.49±6.41 | 13.06±5.09 | 0.986 |
| Unconjugated bilirubin (μmol/L) | 9.15±6.02 | 8.28±5.17 | 0.482 |
| Calcium (mmol/L) | 2.39±0.11 | 2.41±0.12 | 0.757 |
| Potassium (mmol/L) | 4.07±0.35 | 4.1±0.34 | 0.566 |
| Chlorine (mmol/L) | 102.47±3.05 | 103.17±3.05 | 0.145 |
| Sodium (mmol/L) | 140.97±3.49 | 141.19±2.44 | 0.926 |
| CK-MB isoenzyme (U/L) | 14.6±13.87 | 4.8±2.98 | 0.225 |
| CRP (mg/L) | 2.5±8.46 | 2.25±7.12 | 0.204 |
| D-dimer (mg/L) | 0.42±1.03 | 0.26±0.15 | 0.338 |
| INR | 0.98±0.06 | 0.99±0.07 | 0.683 |
| IL-6 (ng/L) | 6.08±14.98 | 4.53±3.23 | 0.743 |
| PCT (μg/L) | 0.04±0.02 | 0.04±0.02 | 0.262 |
| Creatinine (μmol/L) | 60.06±12.36 | 59.52±17.7 | 0.337 |
| Urea (mmol/L) | 5.13±1.26 | 5.17±1.44 | 0.927 |
| Uric acid (μmol/L) | 300.34±88.72 | 318.95±87.96 | 0.225 |
| Glucose (mmol/L) | 5.17±1.95 | 5.57±2.81 | 0.055 |
| Total carbon dioxide (mmol/L) | 26.16±2.94 | 26.42±3.02 | 0.716 |

PRL secreting, prolactin secreting; GH secreting, growth hormone secreting; ACTH secreting, adrenocorticotropic hormone secreting; ICDC4h, the minimum intercarotid distance at the horizontal C4 segment of the internal carotid artery; LH, luteinizing hormone; FSH, follicle-stimulating hormone; DHEAS, dehydroepiandrosterone sulfate; FT3, free triiodothyronine; FT4, free tetraiodothyronine; ACTH, adrenocorticotropic hormone; IGF-1, insulin-like growth factor-1; IGFBP3, insulin-like growth factor binding protein 3; GH, growth hormone; RBC, red blood cell; HCT, haematocrit; RDW, red blood cell distribution width; MCV, mean corpuscular volume; MCH, mean corpuscular hemoglobin; MCHC, mean corpuscular hemoglobin concentration; WBC, white blood cell; MPV, mean platelet volume; TT, thrombin time; PT, prothrombin time; FDP, fibrin/fibrinogen degradation products; ALT, alanine aminotransferase; AST, aspartate transaminase; ALP, alkaline phosphatase; LDH, lactate dehydrogenase; TG, triglyceride; CRP, C-reactive protein; INR, international normalized ratio; IL-6, interleukin-6; PCT, procalcitonin. Tumor signal intensity: T2-weighted magnetic resonance imaging signal intensity of tumor compared with that of white matter.
