# Supplementary material for: Development and Validation of Nomogram to Preoperatively Predict Intraoperative Cerebrospinal Fluid Leakage in Endoscopic Pituitary Surgery: A Retrospective Cohort Study
Source: Front Oncol. 2021 Oct 26;11:719494. doi: 10.3389/fonc.2021.719494 (PMC8576331; doi:10.3389/fonc.2021.719494)
Supplement: Supplementary file 7 [file Table_4.docx]

Supplementary Table 4. Univariate logistic regression analysis of features in the training cohort

| Characteristics | Coefficient | OR | *p* |
| --- | --- | --- | --- |
| Age (year) | 0.0087 | 1.0087 | 0.5510 |
| Gender |  |  |  |
| Female | Reference |  |  |
| Male | -0.6152 | 0.5405 | 0.1020 |
| Primary-recurrence subtype |  |  |  |
| Primary | Reference |  |  |
| Recurrence | 0.874 | 2.3965 | 0.0957 |
| Clinical subtype |  |  |  |
| Non-functioning | Reference |  |  |
| PRL secreting | -1.1192 | 0.3265 | 0.3237 |
| GH secreting | -0.0034 | 0.9966 | 0.9939 |
| ACTH secreting | -0.3953 | 0.6735 | 0.7497 |
| Lengths of tumor maximum dimension (mm) | 0.0573 | 1.059 | 0.0086* |
| Lengths of tumor height (mm) | 0.0768 | 1.0798 | 0.0015* |
| Lengths of tumor width (mm) | 0.0748 | 1.0777 | 0.0202* |
| Lengths of tumor thickness (mm) | 0.1183 | 1.1256 | 0.0017* |
| Tumor volume (mm^3^) | 0.1515 | 1.1636 | 0.0026* |
| ICDC4h | 0.123 | 1.1309 | 0.7080 |
| Hardy grade for suprasellar extension |  |  |  |
| 0 | Reference |  |  |
| A | -0.1515 | 0.8594 | 0.7337 |
| B | -0.2787 | 0.7568 | 0.4948 |
| C | 1.3307 | 3.7837 | 0.0122* |
| D | 1.4018 | 4.0625 | 0.2310 |
| E | 0.9757 | 2.653 | 0.4311 |
| Hardy grade for sellar invasion |  |  |  |
| Noninvasive |  |  |  |
| Invasive | 0.5294 | 1.6979 | 0.2143 |
| Knosp grade |  |  |  |
| Noninvasive |  |  |  |
| Invasive | 0.8109 | 2.2499 | 0.0344* |
| Tumor shape 1 |  |  |  |
| In sella | Reference |  |  |
| Hourglass sign | 0.9671 | 2.6303 | 0.0227* |
| Ellipsoid | -0.6466 | 0.5238 | 0.1492 |
| Tumor shape 2 |  |  |  |
| Not lobulated |  |  |  |
| Lobulated | 1.3116 | 3.7121 | 0.0383* |
| Sellar barrier |  |  |  |
| Weak |  |  |  |
| Strong | -0.8847 | 0.4128 | 0.0427* |
| Tumor signal intensity |  |  |  |
| Lower | Reference |  |  |
| Equal | 0.2282 | 1.2563 | 0.5870 |
| Higher | -0.0736 | 0.929 | 0.8724 |
| Multiple lesions |  |  |  |
| No |  |  |  |
| Yes | 0.2624 | 1.3 | 0.8541 |
| Optic nerve compression |  |  |  |
| No |  |  |  |
| Yes | 0.4447 | 1.56 | 0.2631 |
| Pituitary apoplexy |  |  |  |
| No |  |  |  |
| Yes | 0.8099 | 2.2477 | 0.0523 |
| History of pituitary surgery |  |  |  |
| No |  |  |  |
| Yes | 0.4669 | 1.595 | 0.3336 |
| History of medication |  |  |  |
| No |  |  |  |
| Yes | -5.9575 | 0.0026 | 0.7919 |
| History of radiotherapy |  |  |  |
| No |  |  |  |
| Yes | 6.5443 | 695.2698 | 0.7727 |
| Headache |  |  |  |
| No |  |  |  |
| Yes | 0.0591 | 1.0609 | 0.8735 |
| Visual impairment |  |  |  |
| No |  |  |  |
| Yes | 0.636 | 1.8889 | 0.0955 |
| Visual field defect |  |  |  |
| No |  |  |  |
| Yes | 0.6711 | 1.9564 | 0.0862 |
| Moon face |  |  |  |
| No |  |  |  |
| Yes | 0.2927 | 1.34 | 0.8374 |
| Acromegalia |  |  |  |
| No |  |  |  |
| Yes | 0.4669 | 1.595 | 0.3336 |
| Prolacin (mIU/L) | -2.00E-04 | 0.9998 | 0.4840 |
| Testosterone (nmol/L) | 0.0085 | 1.0085 | 0.8204 |
| Estradiol (pmol/L) | -0.0029 | 0.9971 | 0.2020 |
| Progesterone (nmol/L) | -0.0748 | 0.9279 | 0.2601 |
| LH (IU/L) | -0.0266 | 0.9738 | 0.3007 |
| FSH (IU/L) | -0.001 | 0.999 | 0.9188 |
| DHEAS (umol/L) | -0.0648 | 0.9373 | 0.4902 |
| TSH (mIU/L) | 0.2187 | 1.2445 | 0.0904 |
| T3 (nmol/L) | 0.9006 | 2.4611 | 0.1268 |
| T4 (nmol/L) | 0.0114 | 1.0115 | 0.1504 |
| FT3 (pmol/L) | -0.0493 | 0.9519 | 0.8401 |
| FT4 (pmol/L) | -0.0141 | 0.986 | 0.8402 |
| ACTH (pg/ml) | 0.0096 | 1.0096 | 0.2918 |
| Cortisol (μmol/L) | -0.3073 | 0.7354 | 0.8016 |
| IGF-1 (ng/ml) | 0.001 | 1.001 | 0.5644 |
| IGFBP3 (mg/L) | -0.1972 | 0.821 | 0.1460 |
| GH (μg/L) | 0.005 | 1.005 | 0.8356 |
| RBC count (10^12^/L) | 0.0449 | 1.0459 | 0.9102 |
| HCT (%) | -0.2214 | 0.8014 | 0.9618 |
| RDW (%) | 0.0525 | 1.0539 | 0.7355 |
| MCV (fL) | -0.0058 | 0.9942 | 0.8672 |
| MCH (pg) | -0.0222 | 0.978 | 0.8070 |
| Hemoglobin (g/L) | -0.0011 | 0.9989 | 0.9290 |
| MCHC (g/L) | -0.0011 | 0.9989 | 0.9392 |
| WBC count (10^9^/L) | 0.0834 | 1.087 | 0.4870 |
| Neutrophil percentage (%) | 0.0218 | 1.022 | 0.2410 |
| Lymphocyte percentage (%) | -0.0172 | 0.9829 | 0.3914 |
| Monocyte percentage (%) | -0.2161 | 0.8057 | 0.0785 |
| Basophil percentage (%) | -0.6718 | 0.5108 | 0.4034 |
| Eosinophil percentage (%) | -0.0998 | 0.905 | 0.5015 |
| Platelet count (10^9^/L) | -0.0022 | 0.9978 | 0.5336 |
| Thrombocytocrit (%) | -2.6982 | 0.0673 | 0.4685 |
| MPV (fL) | 0.0537 | 1.0552 | 0.7429 |
| APTT (s) | 0.0933 | 1.0978 | 0.1046 |
| TT (s) | -0.2744 | 0.76 | 0.1228 |
| PT (s) | 0.234 | 1.2636 | 0.3571 |
| Antithrombin III (%) | -0.0033 | 0.9967 | 0.8559 |
| FDP (μg/mL) | -0.2567 | 0.7736 | 0.3676 |
| Fibrinogen (g/L) | 0.6324 | 1.8821 | 0.0510 |
| Total protein (g/L) | -0.0308 | 0.9697 | 0.3869 |
| Albumin (g/L) | -0.0757 | 0.9271 | 0.1779 |
| Globulin (g/L) | -0.0011 | 0.9989 | 0.9841 |
| ALT (U/L) | -1.00E-04 | 0.9999 | 0.9908 |
| AST (U/L) | 0.0204 | 1.0206 | 0.2945 |
| ALP (U/L) | -0.0106 | 0.9895 | 0.3446 |
| LDH (U/L) | -0.0023 | 0.9977 | 0.6021 |
| Total cholesterol (mmol/L) | 0.5527 | 1.7379 | 0.1904 |
| TG (mmol/L) | 0.1137 | 1.1204 | 0.6929 |
| Total bilirubin (μmol/L) | 0.0112 | 1.0113 | 0.7478 |
| Unconjugated bilirubin (μmol/L) | -0.0044 | 0.9956 | 0.9064 |
| Calcium (mmol/L) | 3.9141 | 50.104 | 0.5792 |
| Potassium (mmol/L) | -0.0553 | 0.9462 | 0.9193 |
| Chlorine (mmol/L) | 0.0889 | 1.093 | 0.1923 |
| Sodium (mmol/L) | 0.0219 | 1.0221 | 0.7280 |
| CK-MB isoenzyme (U/L) | -0.1883 | 0.8284 | 0.2003 |
| PCT (μg/L) | 11.6475 | 114405 | 0.3445 |
| CRP (mg/L) | 0.0671 | 1.0694 | 0.4085 |
| D-dimer (mg/L) | -0.5517 | 0.576 | 0.4737 |
| IL-6 (ng/L) | -0.0061 | 0.9939 | 0.9304 |
| INR | 2.4873 | 12.0288 | 0.3935 |
| Creatinine (μmol/L) | -0.002 | 0.998 | 0.8701 |
| Urea (mmol/L) | -0.0295 | 0.9709 | 0.8375 |
| Uric acid (μmol/L) | 0.0037 | 1.0037 | 0.1193 |
| Glucose (mmol/L) | -0.0153 | 0.9848 | 0.9070 |
| Total carbon dioxide (mmol/L) | 0.0735 | 1.0763 | 0.4114 |

PRL secreting, prolactin secreting; GH secreting, growth hormone secreting; ACTH secreting, adrenocorticotropic hormone secreting; ICDC4h, the minimum intercarotid distance at the horizontal C4 segment of the internal carotid artery; LH, luteinizing hormone; FSH, follicle-stimulating hormone; DHEAS, dehydroepiandrosterone sulfate; TSH, thyroid-stimulating hormone; T3, triiodothyronine; T4, tetraiodothyronine; FT3, free triiodothyronine; FT4, free tetraiodothyronine; ACTH, adrenocorticotropic hormone; IGF-1, insulin-like growth factor-1; IGFBP3, insulin-like growth factor binding protein 3; GH, growth hormone; RBC, red blood cell; HCT, haematocrit; RDW, red blood cell distribution width; MCV, mean corpuscular volume; MCH, mean corpuscular hemoglobin; MCHC, mean corpuscular hemoglobin concentration; WBC, white blood cell; MPV, mean platelet volume; APTT, activated partial thromboplastin time; TT, thrombin time; PT, prothrombin time; FDP, fibrin/fibrinogen degradation products; ALT, alanine aminotransferase; AST, aspartate transaminase; ALP, alkaline phosphatase; LDH, lactate dehydrogenase; TG, triglyceride; PCT, procalcitonin; CRP, C-reactive protein; IL-6, interleukin-6; INR, international normalized ratio; OR, odds ratio. Tumor signal intensity: T2-weighted magnetic resonance imaging signal intensity of tumor compared with that of white matter. *Statistical significance.
