# Supplementary material for: Development and Validation of Nomogram to Preoperatively Predict Intraoperative Cerebrospinal Fluid Leakage in Endoscopic Pituitary Surgery: A Retrospective Cohort Study
Source: Front Oncol. 2021 Oct 26;11:719494. doi: 10.3389/fonc.2021.719494 (PMC8576331; doi:10.3389/fonc.2021.719494)
Supplement: Supplementary file 9 [file Table_6.docx]

Supplementary Table 6. Relationship between lengths of tumor height (mm) and some factors

| Characteristics | Lengths of tumor height (mm) | | *p* |
| --- | --- | --- | --- |
|  | Mean | SD |  |
| Knosp grade |  |  | <0.001* |
| Noninvasive | 18.84 | 7.07 |  |
| Invasive | 28.40 | 11.46 |  |
| Hardy grade for suprasellar extension |  |  | <0.001* |
| 0 | 12.40 | 5.28 |  |
| A | 18.63 | 5.56 |  |
| B | 23.68 | 5.17 |  |
| C | 35.27 | 9.81 |  |
| D | 42.54 | 3.38 |  |
| E | 28.11 | 10.21 |  |
| Tumor shape 1 |  |  | <0.001* |
| In sella | 10.54 | 3.01 |  |
| Hourglass sign | 28.60 | 9.36 |  |
| Ellipsoid | 19.48 | 7.09 |  |
| Tumor shape 2 |  |  | 0.017* |
| Not lobulated | 21.54 | 9.24 |  |
| Lobulated | 31.02 | 12.74 |  |
| Sellar barrier |  |  | 0.002* |
| Weak | 27.67 | 11.62 |  |
| Strong | 20.32 | 8.55 |  |

SD, standard deviation. *Statistical significance.
